# Lipopolysaccharide (LPS) potentiates hydrogen peroxide toxicity in T98G astrocytoma cells by suppression of anti-oxidative and growth factor gene expression

**DOI:** 10.1186/1471-2164-9-608

**Published:** 2008-12-16

**Authors:** Gang Yue, Guanfang Shi, Marco A Azaro, Qifeng Yang, Guohong Hu, Minjie Luo, Kingsley Yin, Robert G Nagele, Daniel H Fine, Jin-Ming Yang, Honghua Li

**Affiliations:** 1 Department of Molecular Genetics, Microbiology and Immunology/The Cancer Institute of New Jersey, University of Medicine and Dentistry of New Jersey Robert Wood Johnson Medical School, Piscataway, New Jersey 08854, USA; 2 Department of Chemical Biology, Ernest Mario School of Pharmacy, Rutgers University, Piscataway, NJ 08854, USA; 3 Department of Cell Biology, University of Medicine and Dentistry, NJ-School of Osteopathic Medicine, Stratford, NJ 08084, USA; 4 New Jersey Institute for Successful Aging, University of Medicine and Dentistry of New Jersey School of Osteopathic Medicine, Stratford, NJ 08084, USA; 5 Department of Oral Biology, New Jersey Dental School, University of Medicine and Dentistry, Newark, NJ 07101, USA; 6 Department of Pharmacology, Cancer Institute of New Jersey, New Brunswick, NJ 08901, USA

## Abstract

**Background:**

Lipopolysaccharide (LPS) is a cell wall component of Gram-negative bacteria with proved role in pathogenesis of sepsis. Brain injury was observed with both patients dead from sepsis and animal septic models. However, *in vitro *administration of LPS has not shown obvious cell damage to astrocytes and other relative cell lines while it does cause endothelial cell death *in vitro*. These observations make it difficult to understand the role of LPS in brain parenchymal injury.

**Results:**

To test the hypothesis that LPS may cause biological changes in astrocytes and make the cells to become vulnerable to reactive oxygen species, a recently developed highly sensitive and highly specific system for large-scale gene expression profiling was used to examine the gene expression profile of a group of 1,135 selected genes in a cell line, T98G, a derivative of human glioblastoma of astrocytic origin. By pre-treating T98G cells with different dose of LPS, it was found that LPS treatment caused a broad alteration in gene expression profile, but did not cause obvious cell death. However, after short exposure to H_2_O_2_, cell death was dramatically increased in the LPS pretreated samples. Interestingly, cell death was highly correlated with down-regulated expression of antioxidant genes such as cytochrome b561, glutathione s-transferase a4 and protein kinase C-epsilon. On the other hand, expression of genes encoding growth factors was significantly suppressed. These changes indicate that LPS treatment may suppress the anti-oxidative machinery, decrease the viability of the T98G cells and make the cells more sensitive to H_2_O_2 _stress.

**Conclusion:**

These results provide very meaningful clue for further exploring and understanding the mechanism underlying astrocyte injury in sepsis *in vivo*, and insight for why LPS could cause astrocyte injury *in vivo*, but not *in vitro*. It will also shed light on the therapeutic strategy of sepsis.

## Background

Sepsis is a grave threat to human life in the modern society. It is listed as the second most common cause of death in non-coronary intensive care units and is among the top causes leading to death in the United States [1,2]. The severity of the pathogenesis of sepsis was thought to be the consequence of an uncontrolled hyperinflammatory and mostly cytokine-mediated host response. Recently, a new theory was proposed, which emphasizes on the virulence of microbial pathogens and host-pathogen interactions during severe sepsis [2,3]. A number of extracellular enzymes and microbial mediators have been identified contributing to tissue damage in sepsis. These toxins compromise cellular defenses, cause damage in barriers for microbial invasion, and help the pathogens to spread within the host. In the spectrum of pathogenesis of sepsis, lipopolysaccharide (LPS) has been considered to play a crucial role in pathogen-host interaction [2]. LPS is a major structural component of the outer membrane of Gram-negative bacteria, so as to be often referred as an endotoxin.

Brain injury is observed in postmortem examination of patients dead from sepsis with lesions of multifocal necrotizing leukoencephalopathy, apoptosis, micro-abscesses, and ischemia [4,5]. Systemic LPS administration led to granulocyte influx into brain parenchyma in a mouse model. This influx was accompanied by disruption of the blood-brain barrier to albumin and induction of the intracellular adhesion molecule 1 (ICAM-1) on affected blood vessels [6]. Brain cell death, but no polymorphonuclear infiltration, was also observed in some autopsy materials of patients who died of septic shock [7]. These observations implicate multiple pathways that may underlie the brain cell death process. Brain cell injury could be one of the direct causes leading to septic patient death. For instance, neuronal apoptosis in autonomic centers i.e. cardiovascular autonomic centers indicates that the septic pathogens may set off host mortality by means of damaging host brain cells [4,8].

In the blood-brain barrier, endothelial cells are the first interface interacting with hematogenous spreading LPS. LPS damage to endothelial cells was shown in many studies, one of which showed that LPS induced apoptosis in a bovine endothelial cell line via a soluble CD14 dependent pathway [9]. LPS also induce apoptosis in human endothelial cells [10]. Brain endothelial cell damage during septic shock has also been noticed in clinical patients [11]. LPS triggering brain cell death was observed by de Bock and coworkers [12], who found that LPS endotoxic insult caused neuronal death in cultured organotypic hippocampal slices obtained from 7-day old neonatal rats dependent on the synthesis of tumor necrosis factor alpha (TNF-α) [12]. LPS was also reported to induce death of glial cells in freshly isolated rat neonatal white matter in a dose-dependent fashion [13]. LPS encephalic injection induced endothelial cell and astrocyte injury with increase in blood-brain barrier permeability in rat models [14]. LPS astrocyte injury was also indicated in large animals *in vivo*. Oikawa and coworkers [15] found that septic shock, featured as edema around arterioles and hemorrhages around veins in the brain of horses after systemic administration of LPS [15]. Astrocytes are mainly layered around the blood vessels in the brain. The edema and hemorrhage zones indicate the involvement of astrocytes.

Astrocytes (astroglia) are a subtype of the glial cells in the brain and star-shaped with many functions, including biochemical support of endothelial cells forming the blood-brain barrier, provision of nutrients to the nervous tissue, and a principal role in the repair and scarring process. Astrocytes are the major type of cells around blood vessels in the brain and form the blood-brain barrier with blood vessel endothelial cells. Therefore, in systemically induced or infected endotoxemia, astrocytes will be the direct defense-line of the brain after endothelium being compromised to LPS. When the defensive line of astrocytes is compromised, the brain parenchyma becomes very susceptible to pathogen infection. While LPS causes endothelial cell death both *in vivo *and *in vitro *[14,16,17], astrocyte derivative cell lines such as human cell lines T98G and A172, rat cell lines C6 and immortalized rat astrocytes are broadly used in LPS treated experiments, and no cell injury has been reported [18,19]. If LPS does not cause astrocyte injury *in vitro*, how could LPS cause astrocyte injury *in vivo *in the animal tests? Whether systemic administration of LPS could result in brain parenchyma damage also becomes a question.

Previous publications indicate that LPS alone would not cause cell death, but LPS combined with cytokines, *i.g.*, interferon-γ (INF-γ) would cause decrease in viability of rat C6 and rat primary astrocytes [20,21]. Those reports indicate a much more complex mechanism of LPS on astroctyte injury compared to LPS causing endothelial injury. LPS is involved in broad inflammatory responses. Therefore, a comprehensive study of inflammation and other relevant factors under LPS influence may provide more detailed information about the behavior of astrocytes in sepsis.

The present study was designed to explore LPS role in sepsis in a comprehensive way by profiling expression of a highly selected group of genes using an astrocyte model (T98G). T98G cell line is a derivative of glioblastoma. Its astrocytic origin was confirmed by Bignami *et al. *[22]. The cell line has been intensively used as a model to study astrocytes [23-25] because of its biological resemblance to primary astrocyte. T98G cells express the specific marker of astrocyte, glial fibrillary acidic protein (GFAP) and share other phenotypes to primary astrocyte such as CD68^- ^and HLA-I^- ^[24].

## Results

### LPS potentiates H_2_O_2_-caused cell death

**Figure 1:**
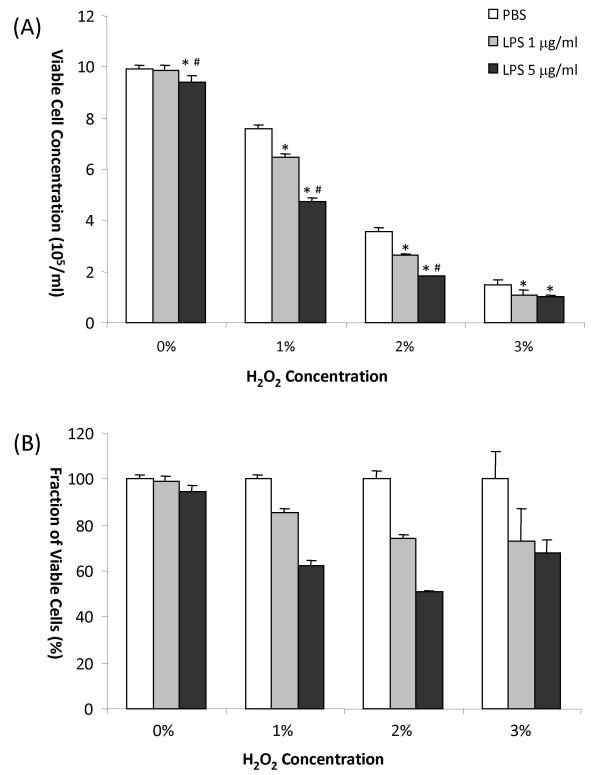
**Illustration of results from the study of astrocyte death caused by LPS treatment followed by H_2_O_2 _exposure**. (A). Effect of LPS and H_2_O_2 _concentration on cell viability. "*" indicates statistically significantly different (α < 0.05) from PBS treatment in the same subgroups (the same H_2_O_2 _concentration), and "#" indicates statistically significantly different (α < 0.05) from sample pretreated with 1 μg/ml LPS in the same subgroup. Vertical bars represent range of variation of the cell viability error bars indexing the standard deviations (SD) based on three independent experiments. (B). The same set of data in (A) but are presented in percentages of viable cells compared with the PBS controls in the same subgroups.

Without subsequent H_2_O_2 _exposure, cellular viability was not altered when cells were treated with 1 μg/ml LPS compared to PBS controls (First panels on the left in Figure 1A and 1B). With higher concentration of LPS (5 μg/ml), it only caused a minor (5%) reduction of cell viability compared to the PBS controls without subsequence H_2_O_2 _exposure (First panels on the left in Figure. 1A and 1B).

By itself, H_2_O_2 _caused substantial cell death, but this effect was greatly enhanced in cells pretreated by LPS (Figure 1). H_2_O_2 _mediated decrease in cell viability was closely related to LPS dose. 1% H_2_O_2 _treatment caused 36% decrease in cell viability among the cells pretreated with 1 μg/ml LPS, while 5 μg/ml LPS caused 50% decrease in cell viability, as compared to the corresponding cells which only treated with LPS without subsequent H_2_O_2 _treatment. In contrast, cell viability only dropped 24% for cells that did not receive LPS beforehand. Similar patterns were obtained in groups treated with 2% and 3% H_2_O_2_.

In all subgroups treated with H_2_O_2_, cell viabilities were significantly reduced (α < 0.05) in the groups pretreated with LPS at as low as 1 μg/ml compared with the cells received PBS instead (Figure 1). The same is true for the groups pretreated with 5 μg/ml LPS versus PBS controls (α < 0.05). With the increase in LPS dosage, a further increased cell death was observed in those cells which received 1 μg/ml LPS or 5 μg/ml LPS and then were exposed to 1% or 2% H_2_O_2 _(Figure 1). For 3% H_2_O_2 _treatments, no significant difference was observed between the two LPS concentrations, which may be because H_2_O_2 _induced cell death became predominant. Those results suggest that LPS pretreatment synergistically enhances the H_2_O_2 _caused cell death.

### LPS induced gene expression changes

**Figure 2:**
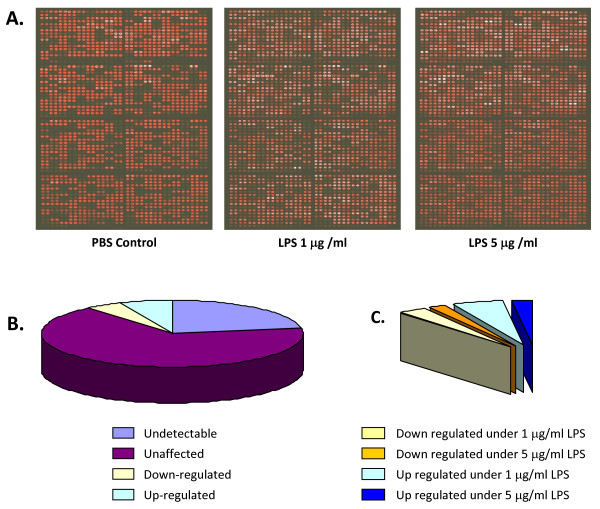
**Illustration of microarray results**. In each array, all the 1,135 oligonucleotide probes were spotted twice. (A). Microarray images for PBS control, 1 and 5 μg/ml LPS treatments; (B) Pie chart presentation of the expression profile of the1135 genes. (C) Illustration of the portion of significantly down- and up-regulated genes in the presence of 1 and 5 μg/ml LPS.

Using multiplex amplification and microarray, we identified 883 (78%) mRNA species with signals significantly higher than backgrounds (*p *< 0.05, Welch's *t*-test) in T98G cells under various conditions (Figure 2). Of the 883 genes, 51 (4.5%) were down-regulated, while 76 (6.7%) were up-regulated by different LPS treatments. In the down-regulated group, the expression of 31 out of 51 genes began to decrease significantly (α < 0.05) at the lower dose of LPS (1 μg/ml), and the rest also showed significant (α < 0.05) down-regulation at higher does LPS (5 μg/ml). In the up-regulated group, 55 out of 76 mRNA species began significantly (α < 0.05) to raise in response to the lower dose of LPS 1 μg/ml, while 21 increased significantly (α < 0.05) at higher dose of LPS (5 μg/ml) (Figure. 2, Additional file 1).

LPS treatments induced alteration in expression for a wide range of genes in the T98G cells, including those relevant to antioxidant, antibacterial, growth factor, apoptosis, gene transcription, brain function, cytoskeletal rearrangement, astrocytic differentiation, transfer of cargo between intracellular membranes of organelles, cell adhesion, cell cycle regulation, pro-inflammatory cytokines, etc. (Additional file 1).

Amidst the group of genes down regulated by LPS, the ones related to antioxidation were outstanding (Additional file 1). These genes included: (1) glutathione S-transferase A-4 (*gsta4*), a member of the glutathione S-transferase family, crucially associated with glutathione antioxidantion [26], (2) kinase C-epsilon (*prkce*) that functions to prevent cell injury from ischemia-reperfusion like insult [27], (3) cytochrome b561 (*cyb561*) that is a ferric reductase to maintain ferrous-ferric homeostasis in cells [28]. Decreased ferrous/ferric ratio was observed in neuronal degeneration diseases [29], indicating the deficiency in the antioxidant in the affected patients, (4) vasoactive intestinal peptide (VIP) type 1 receptor (*vipr1*), which is the major type of receptor mediating VIP activity. VIP which was reported to have antioxidant effect on inhibition of INF-γ stimulated nicotinamide adenine dinucleotide phosphate (NADPH) oxidative pathways in murine macrophages [30], (5) macrophage stimulating protein (*mst1*), which is also known as hepatocyte growth factor-like protein, and was reported to inhibit the production of nitric oxide (NO), a reactive oxygen species, in the injured hypoglossal nuclei [31]. In contrast, endothelial nitric oxide synthase (eNOS) is significantly increased after the LPS treatment. The above data imply a decreased antioxidant capacity in the LPS treated astrocytes.

### Correlation between down-regulated genes and cell viability after H_2_O_2 _treatment

As Pearson product-moment correlation coefficient *r *> 0.988 (α < 0.05) was used as the significance level cutoff, amidst the down-regulated genes, 12 are significantly correlated with cell viability, while only one gene whose expression is significantly correlated with cell viability in the up-regulated group (Table 1).

**Table 1:** Correlation between gene expression and cell viability

**Accession Number**	**Gene Name**	**LPS (μg/ml)**	**Pearson Correlation Coefficient (*r*)**			
			
			**Control (PBS)**	**1% H**_2_**O**_2_	**2% H**_2_**O**_2_	**3% H**_2_**O**_2_
**Down Regulated**						
**Adhesion**						
NM_001797	cadherin 11, type 2, OB-cadherin (osteoblast) (CDH11)	5	0.7715	0.9635	**0.9903**	**0.9993**
**Anti-Oxidant**						
NM_001512	glutathione S-transferase A4 (GSTA4)	1	0.6140	0.8818	0.9360	0.9669
NM_020998	macrophage-stimulating protein (MST1)	1	0.6693	0.9135	0.9589	0.9827
NM_005400	protein kinase C, epsilon (PRKCE)	5	0.6189	0.8847	0.9382	0.9684
NM_004624	vasoactive intestinal peptide receptor 1 (VIPR1)	5	0.7329	0.9462	0.9804	**0.9953**
NM_001915	cytochrome b-561 (CYB561)	5	0.7711	0.9633	**0.9902**	**0.9992**
**Antibacterial Factors**						
NM_020993	B-cell CLL/lymphoma 7A (BCL7A)	1	0.8045	0.9765	**0.9963**	**0.9999**
NM_001735	sapiens complement component 5 (C5)	1	0.5590	0.8478	0.9100	0.9473
NM_000565	interleukin 6 receptor (IL6R)	1	0.6850	0.9220	0.9648	**0.9865**
**Apoptosis**						
NM_000435	notch homolog 3 (Drosophila) (NOTCH3),	1	0.7489	0.9536	0.9848	**0.9973**
NM_001618	poly (ADP-ribose) polymerase family, member 1 (PARP1),	1	0.6838	0.9214	0.9643	0.9862
NM_021136	reticulon 1 (RTN1)	1	0.6122	0.8807	0.9352	0.9663
NM_000041	apolipoprotein E (APOE)	5	0.7164	0.9381	0.9754	0.9927
NM_001964	early growth response 1 (EGR1)	5	0.7975	0.9739	**0.9952**	**1.0000**
**Brain Function**						
NM_000027	aspartylglucosaminidase (AGA)	1	0.5997	0.8732	0.9295	0.9621
NM_000441	solute carrier family 26, member 4 (SLC26A4)	5	0.7566	0.9571	0.9868	0.9981
NM_004283	RAB3D, member RAS oncogene family (RAB3D)	5	0.8235	0.9830	**0.9986**	**0.9988**
NM_003045	solute carrier family 7 (cationic amino acid transporter, y+ system), member 1 (SLC7A1)	5	0.6901	0.9247	0.9666	0.9876
**Cancer Marker**						
NM_016730	folate receptor 1 (adult) (FOLR1)	1	0.6441	0.8994	0.9489	0.9760
NM_005635	synovial sarcoma, X breakpoint 1 (SSX1)	1	0.7612	0.9591	0.9879	**0.9985**
**Cell Cycle Regulation**						
NM_005982	SIX homeobox 1 (SIX1)	5	0.6397	0.8969	0.9471	0.9748
NM_005994	T-box 2 (TBX2)	5	0.6035	0.8755	0.9312	0.9634
**Cytoskeletal Constitution and Rearrangement**						
NM_001621	aryl hydrocarbon receptor (AHR)	1	0.6673	0.9124	0.9582	0.9822
NM_000269	non-metastatic cells 1, protein (NM23A) expressed in (NME1)	1	0.5921	0.8685	0.9260	0.9595
NM_003289	tropomyosin 2 (beta) (TPM2)	1	0.7030	0.9314	0.9710	**0.9903**
NM_015873	villin-like (VILL)	1	0.5310	0.8296	0.8956	0.9361
NM_003023	SH3-domain binding protein 2 (SH3BP2)	5	0.7448	0.9517	0.9837	**0.9969**
**Extra Cellular Matrix-Degrading Proteases**						
NM_007038	ADAM metallopeptidase with thrombospondin type 1 motif, 5 (aggrecanase-2) (ADAMTS5)	1	0.6070	0.8776	0.9328	0.9646
**Growth Factor and Related Protein Factors**						
NM_018127	elaC homolog 2 (E. coli) (ELAC2)	1	0.6819	0.9203	0.9636	0.9858
NM_003506	frizzled homolog 6 (Drosophila) (FZD6),	1	0.7105	0.9352	0.9735	**0.9917**
NM_018344	solute carrier family 29 (nucleoside transporters), member 3 (SLC29A3)	1	0.6177	0.8840	0.9376	0.9680
NM_021724	nuclear receptor subfamily 1, group D, member 1 (NR1D1)	1	0.6802	0.9194	0.9630	0.9854
NM_001012	ribosomal protein S8 (RPS8)	1	0.6111	0.8801	0.9347	0.9659
NM_003087	synuclein, gamma (breast cancer-specific protein 1) (SNCG)	1	0.7776	0.9660	0.9916	**0.9996**
NM_015906	tripartite motif-containing 33 (TRIM33),	1	0.7008	0.9303	0.9703	**0.9898**
NM_000222	v-kit Hardy-Zuckerman 4 feline sarcoma viral oncogene homolog (KIT)	1	0.6613	0.9091	0.9558	0.9807
Y12863	growth factor FIGF	5	0.7831	0.9683	**0.9927**	**0.9998**
NM_002086	growth factor receptor-bound protein 2 (GRB2)	1	0.6311	0.8919	0.9434	0.9722
M35410	insulin-like growth factor binding protein 2 (IGFBP2)	1	0.6801	0.9194	0.9630	0.9854
NM_001050	somatostatin receptor 2 (SSTR2)	1	0.9252	**0.9996**	0.9871	0.9658
NM_016135	ets variant gene 7 (TEL2 oncogene) (ETV7)	5	0.6810	0.9198	0.9633	0.9856
NM_006732	FBJ murine osteosarcoma viral oncogene homolog B (FOSB)	5	0.6889	0.9240	0.9662	0.9873
NM_022963	fibroblast growth factor receptor 4 (FGFR4)	5	0.6720	0.9150	0.9600	0.9834
NM_002957	retinoid × receptor, alpha (RXRA)	5	0.9264	**0.9995**	0.9866	0.9650
NM_004630	splicing factor 1 (SF1)	5	0.8712	**0.9955**	**0.9993**	**0.9904**
NM_005252	v-fos FBJ murine osteosarcoma viral oncogene homolog (FOS)	5	0.8257	0.9837	**0.9988**	**0.9986**
**Hemostasis**						
NM_000624	peptidase inhibitor, clade A (alpha-1 antiproteinase, antitrypsin), member 5 (SERPINA5)	1	0.6092	0.8790	0.9339	0.9653
**Inflammation and Immunity**						
NM_000963	cyclooxygenase-2 (COX2)	1	0.5186	0.8213	0.8890	0.9308
**Transfer of Cargo Between Intracellular Membrane Organelles**						
NM_016577	RAB6B, member RAS oncogene family (RAB6B)	1	0.7002	0.9299	0.9701	**0.9897**
**Up Regulated**						
**Cancer Marker**						
NM_005814	glycoprotein A33 (transmembrane) (GPA33)	1	-0.9658	-0.9878	-0.9589	-0.9251
**Cell Junction**						
NM_001943	desmoglein 2 (DSG2)	1	-0.6663	-0.9119	-0.9578	-0.9820
**Cytoskeletal Constitution and Rearrangement**						
NM_002613	3-phosphoinositide dependent protein kinase-1 (PDPK1)	1	-0.5869	-0.8653	-0.9235	-0.9577
**Gene Transcription**						
NM_006180	neurotrophic tyrosine kinase receptor, type 2 (NTRK2)	1	-0.6822	-0.9205	-0.9637	-0.9858
NM_013275	nasopharyngeal carcinoma susceptibility protein; lz16/ankyrin repeat domain 11 (ANKRD11)	1	-0.7789	-0.9666	**-0.9918**	**-0.9997**

*Genes with a Pearson's product-moment correlation coefficient (*r*^2^) > 0.85 are listed. Those with coefficient *r *> 0.988 (α < 0.05) are in bold face.

Interestingly, the *r *values for cytochrome b561, the ferric reductase, are 0.771, 0.963, 0.990, and 0.999 in the groups treated with PBS, 1%, 2% and 3% H_2_O_2_, respectively (Table 1), indicating a strong intrinsic association of cytochrome b561 gene expression and cell viability. VIP receptor 1 displays an *r *of 0.995 in the 3% H_2_O_2 _treated group (Table 1). The other antioxidant genes also had high *r *values although are not significant. For instance, *r *values for *gsta4*, *prkc*, and *mst1 *are 0.967, 0.968, and 0.983 in the 3% H_2_O_2 _treated groups, respectively (Table 1).

Other impressive genes with their expression highly correlated with cell viability are growth factors, including *c-fos *induced growth factor (*figf*), growth factor receptor-bound protein 2 (*grb2*), insulin-like growth factor binding protein 2 (*igfbp2*), v-fos FBJ murine osteosarcoma viral oncogene homolog (*fos*) and FBJ murine osteosarcoma viral oncogene homolog B (*fosb*). (Table 1, and Additional file 1). The expression of *figf *and *fos *achieved a significant level to correlate with cell viability, while *fosb*, *grb2 *and *igfbp2 *show high, but not significant *r *values of 0.987, 0.972 and 0.985, respectively, in the 3% H_2_O_2 _group (Table 1). The gene expressions of growth factor receptor-bound protein 2 (*grb2*) and insulin-like growth factor binding protein 2 (*igfbp2*) also showed high correlation with T98G cell viability (Table 1). The gene products of those two genes are involved in cross-talking of growth factor pathway and other pathways [32-34]. Decline of somatostatin receptor 2 (*sstr2*) gene expression was reported to be associated with brain cell injury [35]. In the present study, *sstr2 *gene expression showed significant correlation with T98G cell viability in 1% H_2_O_2 _(Table 1). Nerve growth factor was detected in the present study, but its gene expression appeared not to be affected by LPS treatment (Table 2), implying nerve growth factor may not be involved in causing astrocyte death in sepsis at the gene expression level.

**Table 2:** Genes associated with cytokines, cell death, and growth

**Accession Number**	**Gene Name**	mRNA*
**Interleukins**		
NM_000576	interleukin beta; il1 b	U/A
M14584	interleukin 6 (interferon; beta 2); IL-6	U/A
NM_000882	interleukin 12a; il12a	U/A
NM_000641	interleukin 11; il11	Up
NM_000640	interleukin 13 receptor alpha 2; il13ra2	Up
NM_000565	interleukin 6 receptor; IL-6Ra	Down
NM_000586	interleukin 2; il2	U/D
NM_000588	interleukin 3 colony-stimulating factor multiple; il3	U/D
NM_000589	interleukin 4; il4	U/D
NM_000879	interleukin 5 colony-stimulating factor eosinophil; il5	U/D
NM_000590	interleukin 9; il9	U/D
NM_002188	interleukin 13; il13	U/D
NM_001562	interleukin 18; il18	U/D
**Tumor Necrosis Factors**		
NM_003807	tumor necrosis factor ligand superfamily member 14; tnfsf14	Up
NM_001065	tumor necrosis factor receptor 55kd; tnfrsf1a	U/A
NM_001066	tumor necrosis factor receptor 2 75kd; tnfrsf1b	U/A
NM_006291	tumor necrosis factor alpha-induced protein 2; tnfaip2	U/A
NM_032945	tumor necrosis factor receptor superfamily member 6b precursor isoform d; tnfrsf6b	U/A
NM_003811	tumor necrosis factor ligand superfamily member 9; tnfsf9	U/A
NM_003810	tumor necrosis factor ligand superfamily member 10; tnfsf10	U/A
NM_003842	tumor necrosis factor receptor superfamily member 10b; tnfrsf10b	U/A
NM_006573	tumor necrosis factor ligand superfamily member 13b; tnfsf13b	U/A
NM_003820	tumor necrosis factor receptor superfamily member 14; herpesvirus entry mediator; tnfrsf14	U/A
NM_000594	tumor necrosis factor cachectin (TNF)	U/D
NM_001244	tumor necrosis factor ligand superfamily member 8; tnfsf8	U/D
NM_003844	tumor necrosis factor receptor superfamily member 10a; tnfrsf10a	U/D
**Apoptosis**		
U82987	Bcl-2 binding component 3 (bbc3)	Up
NM_003879	casp8 and fadd-like apoptosis regulator; cflar	U/A
NM_032977	caspase 10 isoform d small subunit; casp10	U/A
NM_033356	caspase 8 isoform c; casp8	U/A
NM_000043	apoptosis apo-1 antigen 1; tnfrsf6	U/A
NM_001160	apoptotic protease activating factor isoform b; apaf1	U/A
U59747	BCL2-like 2; Bcl-w	U/A
NM_032989	bcl2-antagonist of cell death protein; bad	U/A
AF022224	Bcl-2-associated athanogene; BAG-1	U/A
NM_003766	beclin coiled-coil myosin-like bcl2-interacting protein; becn1	U/A
NM_000639	apoptosis apo-1 antigen ligand 1; tnfsf6	U/D
NM_021631	apoptosis inhibitor; fksg2	U/D
NM_004874	bcl2-associated athanogene 4; bag4	U/D
**Nerve Growth Factors**		
NM_002507	nerve growth factor receptor; ngfr	U/A
X52599	nerver growth factor; beta polypeptide; NGF	U/A

*U/A, unaffected. U/D, undetectable

The present study indicates that astrocytes may not be the major source to release chemotactic factors, and therefore, they may not actively recruit monocytes and neutrophils to the site under LPS treatment. Our results indicate that gene expression of *il1b*, *il6 *and *il12 *were suppressed by LPS, while mRNAs *of il2*, *il3*, *il4, il5, il9 *and *il13 *were not detectable in T98G cells (Table 2). *il11 *expression was increased by LPS treatment (Table 2), which is consistent to the notion that *il11 *is expressed in astrocytes under inflammatory stimuli [36]. Interestingly, the *il11 *generated by astrocytes in response to inflammation may have a protective function for survival of oligodendroglia that myolinated the axons of neurons [36]. The LPS induced *il11 *gene expression may reflect a cell protective mechanism in sepsis.

Several tumor necrosis factor (*tnf*) family members were included in the present study. Gene expression of *tnfα*, the most studied cytokine involved in inflammation and cell death, was not detectable in T98G cells, while gene expression of its receptors, *tnfr1 *and *tnfr2 *were unaffected by the LPS treatment, indicating cell death induced in the present study was independent of gene expressions of *tnfα*, *tnfr1 *and *tnfr2 *of T98G (Table 2). Gene expression of TNF ligand superfamily member 14 (*tnfsf14*) was increased as the LPS dose increased, but did not show meaningful correlation with the cell viability (Table 2). TNFSF14 which is a homolog of lymphotoxin was reported to inhibit tumor growth [37]. Considering the cancerous nature of T98G cell, LPS induced *tnfsf14 *expression may imply brain tumor suppression function in some cases [38]. Conspicuously, cyclooxygenase 2 (*cox2*) gene expression was significantly suppressed by LPS pretreatment (Table 1, and Additional file 1), though the suppression level was not significantly correlated with cell viability. The gene of *cox2 *is broadly expressed in cancers and is suggested to be a potent enzyme in arachidonic acid metabolism to favor the tumor growing [39]. Our results are in consistent with the anticancerous effect of LPS [38].

With the undetectable or unaffected mRNAs of TNFα and its receptors under LPS treatment, caspase 8 and caspase 10, the key factors to mediate apoptotic signals from the TNF family [40], was also unaffected by the LPS in their gene expression (Table 2). *Bcl-2 *family plays crucial roles in pro- or anti-apoptosis [41]. Neither mRNA of *Bcl-w*, a factor functioning in blocking apoptosis, nor mRNA of Bad, the factor playing an opposite role as to *Bcl-w*, was affected by LPS (Table 2). Markedly, gene expression of *Bcl-2 *binding component 3 (*bbc3*), a strong pro-apoptotic factor, was induced by LPS (Table 1, 2). The *bbc3 *gene is a direct target of p53 and is also induced by p53 independent apoptotic stimuli such as dexamethason treatment and serum deprivation [42]. Interestingly, its expression could be suppressed by growth factors [42], which may coordinate the growth factor suppression in the present study. The *bbc3 *gene expression does not show a correlation with the cell death, indicating the possibility of multiple regulation levels involved in the cell death resulted from LPS coupled with H_2_O_2 _treatment.

## Discussion

Our results showed that LPS itself was insufficient to cause T98G cell death. However, when subsequently treated with H_2_O_2_, the LPS effect in inducing cell death could be significantly enhanced, which might be underlain by altered gene expression through pre-exposure of LPS. In systemic sepsis, LPS first accesses the endothelial layer of the blood-brain barrier. In addition to cell death leading to compromise integrity of endothelial layer in the blood-brain barrier, the permeability of the vascular barrier can also be changed in response to LPS via paracelluar permeability. The paracellular pathway is composed of both tight junctions and adherens junctions between endothelial cells. These inter-endothelial junctions are compromised under LPS stimulation and leaky to liquid and solutes [43]. In this case, astrosytes are exposed to LPS directly.

LPS treatment suppressed expression of antioxidant genes such as *cyb561*, *gsta4*, *prkce*, *mst1 *and *vipr1 *(Table 1, and Additional file 1). Suppression of antioxidant gene expression subjects the cells to increased oxidative stress. When this happens in astrocytes, LPS may simultaneously stimulate neighboring cells, i.e., endothelial cells to release chemotactic factors such as TNFα, IL-1β, INF-γ to attract monocytes and nerutrophils to the niche. Subsequently, monocytes and neutrophils are triggered by LPS and proinflammatory cytokines to generate H_2_O_2_. In the brain, microglial cells are the member of the monocyte/macrophage family and it may be activated by LPS to generate H_2_O_2 _in order to destroy invading bacteria but may cause injury to brain tissues as well [44]. The coexistence of microglia may explain LPS induced cell death in cultured brain slices and in freshly isolated white matter glial cells [12,13].

Cyt b561, GSTA4 and PRKCE are not directly associated with NO pathways, though an increase in NO generation was observed under LPS stimulation in macrophage/monocyte [45], neutrophil [46] and even astrocyte [47,48] via inducible nitric oxide synthase (iNOS). LPS as an exogenous inducing factor to iNOS has long been established. Therefore, iNOS was not included in the present study. The scavenging of H_2_O_2 _may reduce the tissue level of superoxide (O_2_^.^) on a stoichiometric basis because it is produced from O_2_^. ^catalyzed by superoxide dismutase. It is plausible that a reduction of tissue O_2_^. ^may reduce the production of peroxinitrite that is produced by interaction of O_2_^. ^and NO and is a major toxic metabolite of NO causing cell injury [49]. Moreover, MST1 is known to inhibit the production of NO [31]. Its gene expression is also down regulated by the LPS with an *r *value of 0.98 correlated with cell death in the present study, indicating that a weakened defensiveness to NO and its toxic metabolites may also occur in astrocyte under LPS stress.

Furthermore, the present study showed that LPS suppressed gene expression of growth factors and associated factors. Among the suppressed genes, gene product of *figf *was reported to be a survival factor in human cell lines by increasing Bcl-2 expression, decreasing caspase activities and inhibition of poly(ADP-ribose) polymerase cleavage to resist hypoxia and chemical induced cell death [50]. *fos *is a cellular proto-oncogene belonging to the immediate early gene family of transcription factors in response to growth factor and other stimuli [51]. The *fos *gene family is comprised of four members: *fos, fosb, fosl1*, and *fosl2*, which encode leucine zipper proteins that can dimerize with proteins of the JUN family, thereby forming the transcription factor complex AP-1, regulating cell proliferation, differentiation, and transformation [52]. FosB encoded by *fosb *acts as a regulator in cell proliferation, differentiation, and transformation [51]. GRB2 is a plasma protein involved in mediating growth factor signals via promoting growth factor induced growth-receptor endocytosis [53] and interacting with Ras as well as p21-activated kinase 1 (PAK1) to weave the growth factor pathways [33,34]. IGFBP2 was reported to play a key role in driving glioma cell growth via activation of the Akt pathway and to collaborate with K-Ras or platelet-derived growth factor β in the development and progression of glioma [32]. In consistent with the previous studies that expression of *sstr2 *declined in brain cells in rat trauma model and was associated with brain cell injury [35], the *sstr2 *gene expression displayed significant correlation with cell viability in 1% H_2_O_2 _in our present study (Table 1). Since *sstr2 *was reported to have neuroprotective potential [35], suppression of *sstr2 *gene expression may also contribute to astrocyte death in the present study.

The present study outlines a rational profile on the possible mechanisms that LPS compromises the blood-brain barrier during sepsis. Suppression of antioxidant gene expression of *cyb561*, *gsta4*, and *prkce *indicates a reduction of cell antioxidative capacity. Furthermore, the co-suppression of growth factors and related factors such as *figf, fos, fosb, grb2 *and *igfbp2 *may further weaken the cell survival ability to resist harsh stress such as that caused by H_2_O_2_. Growth factors are involved in broad functions in maintaining cell survive. In addition to regulate Bcl-2 and caspase activities, and inhibition of poly(ADP-ribose) polymerase cleavage [50], growth factor may also promote glutathione redox cycling. For instance, nerve growth factor has potent effect to resist H_2_O_2 _damage on neurons via a rapid activation of glutathione redox cycling [54], indicating the direct effect of growth factor to protect cells from reactive oxygen species. Interestingly, in the present study, gene expression of nerve growth factor was not affected by LPS (Table 2), indicating gene expression of nerve growth factor may not be an LPS target to induce the astrocyte death.

## Conclusion

The facts of LPS suppression of genes encoding antioxidant factors and growth factors, and H_2_O_2 _enhancement of the LPS pretreated cell death suggest a possible crucial mechanism causing blood-brain barrier damage in sepsis and at least in part explains the previously described discrepancy why LPS cause brain cell injury *in vivo *or in organ culture, but not remarkable with culture astrocytes. When LPS is systemically administered, monocytes and neutrophils may be recruited into the inflammatory niche. These cells may subsequently, generate H_2_O_2 _to trigger cell death. In brain slice organ culture, in addition to the possibly remaining monocytes and macrophages, microglial cells could be the principal source to generate reactive oxygen species in the presence of LPS [55]. These results may shed light on the therapeutic strategies in septic shock patients, though further studies including *in vivo *studies are needed to define the detailed pathways as well as regulations at the translation and protein-function levels in mediation of astrocyte death.

## Methods

### Cell culture, and LPS and H_2_O_2 _treatments

T98G was purchased from American Type Culture Collection (Manassas, VA). The cell line was maintained in Ham's F-10/DMEM (10:1) medium supplemented with 10% fetal bovine serum, 100 units/ml penicillin, and 100 μg/ml streptomycin at 37°C in a humidified atmosphere containing 5% CO_2_/95% air. When 80% confluence was achieved, LPS (Salmonella enteriditis, Sigma-Aldrich) was added to the final concentrations of 1 and 5 μg/ml, respectively. The controls received equal volumes of PBS instead. After 24-hr treatment with LPS, cells were washed with PBS for three times. Serum free media were added into the cell cultures. Thirty percent of H_2_O_2 _was then added into cell cultures to final concentrations of 1%, 2% and 3%, respectively. Control groups received PBS with no H_2_O_2 _instead. After one hour exposure, H_2_O_2 _was washed away with PBS for three times. Cells were re-cultured in regular media for extra 24 hrs. Cell viability was measured with MTT assay as instructed by the vender (Promega). Data were obtained from three independent experiments.

### Primer and probe design

Our recently developed highly sensitive and specific system for high-throughput gene expression profiling was employed [56-58] to study the gene expression profiles in the T98G cells under different experimental conditions. Compared with many conventional methods, the major advantage of this technology is its ability to detect mRNA and to amplify cDNA without using poly-A tails. This system is especially advantageous for the present study since missing Poly-A tails is a remarkable feature in brain mRNA. Our system uses specially designed primer pairs crossing introns for multiplex PCR to make the amplification highly specific. The detection specificity is further guaranteed by using an oligonucleotide probes consisting of sequences in two neighboring exons for microarray detection. With a special primer selection strategy, over 1,000 mRNA species can be detected by a single assay [56,58].

A panel of 1,135 mRNA species relevant to inflammation, cell growth, differentiation, apoptosis, gene expression, antioxidant, *etc. *was amplified simultaneously using our high-throughput system. For each dose of LPS treatment and the PBS control, data were obtained from two separate cell cultures of the same cell line, T98G. Each culture was analyzed with duplicated microarrays. In each array, all oligonucleotide probes were spotted twice. Therefore, each treatment was totally repeated eight times. For each array, 57 mismatch probes were selected from human genome as hybridization controls, which showed no interaction with the genes for testing.

The two conventional housekeeping gene controls, glyceraldehyde-3-phosphate dehydrogenase (GAPDH) and β-actin were used as controls in the present study. Endothelial nitric oxide synthase 3 (NOS3) was used as an additional control since both GAPDH and β-actin gene share significant sequence identity with many pseudogenes. NOS3 is constitutively expressed and may be inducible in the brain. It has been known that it has no splicing alternative and pseudogene.

### One-Step RT-PCR

For One-Step RT-PCR, 200 T98G cells were used after cell lysis with a freezing and thawing procedure [56-58]. Briefly, cells in the lysis buffer (1.5 μl RNasin^® ^Ribonuclease inhibitor, 2 μl of 5× QIAGEN OneStep RT-PCR buffer, 5.5 μl H_2_O) were lysed with three repeating cycles of alternating one-min incubation between an ethanol/dry ice mix and a 37°C water bath. One-step RT-PCR was carried out in a 50-μl reaction containing primers (20 nM each) for all the 1,135 mRNA species, 2.5 mM MgCl_2_, the four dNTPs (400 μM each), 5 units of RNasin^® ^Ribonuclease Inhibitor (Promega) and 2.0 μl QIAGEN OneStep RT-PCR Enzyme Mix. The samples were first treated at 50°C for 40 min to synthesize the selected cDNAs, and then were heated to 95°C for 15 min to inactivate the reverse transcriptase and activate the *Taq *DNA polymerase followed by 35 PCR cycles. Each PCR cycle consisted of 40 sec at 94°C for denaturation, and 1 min at 50°C for annealing and 5 min of ramping from 50°C to 70°C for annealing and extension. At the end of the PCR, a final extension step was carried out at 72°C for 3 min. All PCRs were performed with the PTC100 Programmable Thermal Controllers (MJ Research). Single-stranded DNA (ssDNA) was generated by using the same conditions in the multiplex PCR step except for the templates that were 2 μl of the multiplex RT-PCR product. Only one primer for each sequence was used, and 50 thermal cycles were carried out.

### Microarray hybridization and probe labeling

Hybridization was performed in 30 μl of 1× hybridization solution (5× Denhart's solution, 0.5% SDS, 3 × SSC, 20 μl of ssDNA at 56°C for 2 hrs. The slide was then washed with 1 × SSC and 0.1% SDS at 56°C for 10 min, rinsed twice with 0.5 × SSC for 30 sec and twice with 0.2 × SSC for 30 sec. Microarrays were covered with 25 μl 1× labeling solution containing 20 units of Sequenase, 1× Sequenase buffer (GE Healthcare Life Sciences), and 750 nM Cy5-ddCTP (Applied Biosystems). The labeling reaction was performed at 70°C for 10 min. The slide was washed again under the same conditions used after hybridization.

### Microarray scan

Microarrays were scanned with a GenePix 4000 scanner (Axon Instruments, Foster City, CA). The resultant images were digitized with the accompanying software GenePix Pro (version 4.0). Cross-array normalization was performed using the linear method described by Hansson *et al.*, [59].

### Data analysis

Signal intensity of each spot was transformed into its natural logarithm. The two-sample *t*-test, Welch's *t*-test, which was considered as an appropriate test for analyzing microarray data [60] was used in the present study. Normalized intensities of each gene were compared to the intensities of the mismatch probes as described by Bonaventure *et al. *[61]. A gene is considered expressed if its signal intensity is significantly greater than controls with a *p*-value < 0.05 to reject the null hypothesis in all three treatments including PBS, 1 μg/ml and 5 μg/ml LPS treatments.

The One-way ANOVA followed by Bonferroni Correction Test was used to determine the gene expression changes in response to the LPS treatments. For each mRNA species the above three treatments were compared. A gene was considered as down regulated if the means of signal densities of these three treatments were in an order of PBS > 1 μg/ml LPS > 5 μg/ml LBS, and at least one of the comparisons between the three treatments reached a significance level of α < 0.05 in the One-Way ANOVA Test followed by Bonferroni Correction Test. If the results were in a reversed order, the gene was considered as up-regulated. The effect of gene expression levels on cell viability was also studied. For each gene, the gene expression levels of the three treatments were compared with cell viabilities in pair with corresponding groups, i.e. the gene expression level of the group treated with 1 μg/ml LPS was paired with the cell viability of the group received 1 μg/LPS treatment and so on. The relationship between those two variables, gene expression level and cell viability, were examined by the Pearson's Correlation. Pearson product-moment correlation coefficient *r *> 0.988 (α < 0.05) was used as an index for determining whether gene expression has effect on cell viability. In the gene expression down-regulated group, decreased cell viability indicated a positive correlation. *Vice versa*, a negative correlation was ascertained if decreased cell viability was observed in the up-regulated group.

## Authors' contributions

GY conceived of the study, carried out data collection and analysis, and made major effort in manuscript preparation. GS collected and analyzed the T98G cell viability and other data, and conceived of critical ideas for the study. MA developed computer programs for data analysis. QY and GH designed the high-throughput gene expression profiling panel and made microarrays. GH also did microarray data extraction. ML helped to initiate the study. KY and RN conceived of idea for project initiation and other intellectual interaction during the study, and manuscript preparation. DF conceived ideas about the experimental design and data analysis. JY carried out T98 cell culture and treatment. HL conceived of the idea of the high-throughput gene expression profiling experimental scheme, manuscript preparation, and oversaw the study. All authors read and approved the final manuscript.

## Supplementary Material

Additional file 1 **The 127 genes with altered gene expression in the T98G cells after treatment with LPS**. The 127 genes that were up and down regulated in the T98G cells after LPS treatment. Click here for file
